# Polymorphism related to cardiovascular risk in hemodialysis subjects: a systematic review

**DOI:** 10.1590/2175-8239-JBN-3857

**Published:** 2018-06-18

**Authors:** Karla Pereira Balbino, Helen Hermana Miranda Hermsdorff, Josefina Bressan

**Affiliations:** 1 Universidade Federal de Viçosa, Departamento de Nutrição e Saúde, Viçosa, MG, Brasil.

**Keywords:** Chronic Renal Failure, Inflammation, Oxidative Stress, Vascular Calcification, Hypertrophy, Left Ventricular, Polymorphism Single Nucleotide, Insuficiência Renal Crônica, Inflamação, Estresse Oxidativo, Calcificação Vascular, Hipertrofia Ventricular Esquerda, Polimorfismo de Nucleotídeo Único

## Abstract

Cardiovascular disease (CVD) is one of the leading causes of mortality in hemodialysis (HD) subjects. In addition to the traditional risk factors that are common in these individuals, genetic factors are also involved, with emphasis on single nucleotide polymorphs (SNPs). In this context, the present study aims to systematically review the studies that investigated the polymorphisms associated with cardiovascular risk in this population. In general, the SNPs present in HD individuals are those of genes related to inflammation, oxidative stress and vascular calcification, also able of interfering in the cardiovascular risk of this population. In addition, polymorphisms in genes related to recognized risk factors for CVD, such as dyslipidemia, arterial hypertension and left ventricular hypertrophy, also influence cardiovascular morbidity and mortality.

## RESUMO

A doença cardiovascular (DCV) é uma das principais causas de mortalidade de indivíduos em hemodiálise (HD). Além dos fatores de risco tradicionais, que são frequentes nesses indivíduos, também estão envolvidos fatores genéticos, com destaque para os polimorfismos de nucleotídeo único (do inglês, single nucleotide polymorphism, SNP). O presente trabalho tem como objetivo revisar sistematicamente os estudos que investigaram os polimorfismos associados ao risco cardiovascular nessa população. De modo geral, os SNPs presentes em indivíduos em HD são aqueles de genes relacionados à inflamação, estresse oxidativo e calcificação vascular, também capazes de interferir no risco cardiovascular dos pacientes. Polimorfismos em genes relacionados a fatores de risco reconhecidos para DCV, como dislipidemia, hipertensão arterial e hipertrofia ventricular esquerda, também influenciam a morbidade e mortalidade cardiovascular.

## INTRODUCTION

Chronic Kidney Disease (CKD) is defined as a renal parenchyma lesion and/or decreased glomerular filtration rate (less than 60 ml/min/1.73 m^2^), present for a period of three months or more, with health implications.^1^ This disease has high incidence and prevalence rates, and it is a global health problem, with high public healthcare costs of approximately R$ 1.4 billion/year in Brazil.^2^

Epidemiological studies in Brazil have registered a gradual increase in the number of patients with CKD, with a high prevalence rate of dialysis treatments, which served 112.004 patients in 2014. Of these, 91% were on hemodialysis (HD).^3^

Despite improvements in dialysis technology, the mortality rate of HD patients is very high, the main cause of which is cardiovascular disease (CVD). Although traditional risk factors such as hypertension, diabetes *mellitus*, dyslipidemia, age, and smoking are common in these individuals, they only account in part for the high prevalence of CVD. As in other multifactorial diseases, it is suggested that genetic factors are involved in its pathogenesis.^4^

Within this context, several single nucleotide polymorphisms (SNPs), characterized by the variation of a single base pair in the DNA sequence, have been identified in HD individuals.^5^ Some SNPs lead to amino acid substitution in proteins and others cause the production of stop codons, prematurely interrupting protein translation processes, both capable of interfering with its biological function.^6^ Thus, some studies demonstrate the influence of these SNPs on cardiovascular risk in HD individuals.^7^^-^ 9

The aim of this paper is to systematically review studies investigating the polymorphisms associated with cardiovascular risk in HD individuals.

## METHODOLOGY

This systematic review was conducted according to a specific protocol, and it is described according to the items of preferential reports for systematic review and meta-analyzes statement.^10^ This paper is based on previous studies and does not involve studies by any of the authors.

### SEARCH STRATEGY

We conducted a literature review in the MEDLINE (PubMed), Latin American and Caribbean Literature in Health Sciences (LILACS) and Science Direct databases, using the keywords "hemodialysis", "end-stage renal disease" , "Renal replacement therapy", "ESRD", combined with "polymorphism" OR "polymorphisms". The research was limited to articles published between 2010 and 2017.

### SELECTION CRITERIA

Our analysis included clinical studies with adults and elderly undergoing HD treatment. Articles that were not published in full or those presented as tutorials, editorials, news, letters or comments, narrative and systematic reviews, meta-analyzes, case studies, experimental essays, original studies with different themes of interest and repetitions were excluded. Also excluded were studies on acute kidney disease, CKD in conservative treatment or treatment with peritoneal dialysis, transplantation, and nephrotic syndrome.

### SEARCH RESULTS

The studies identified in the electronic databases were gathered into a single database to exclude duplicates. After the exclusion of all duplications, two independent reviewers selected the references in three phases: title analysis, abstracts and full texts.

**Figure 1:**
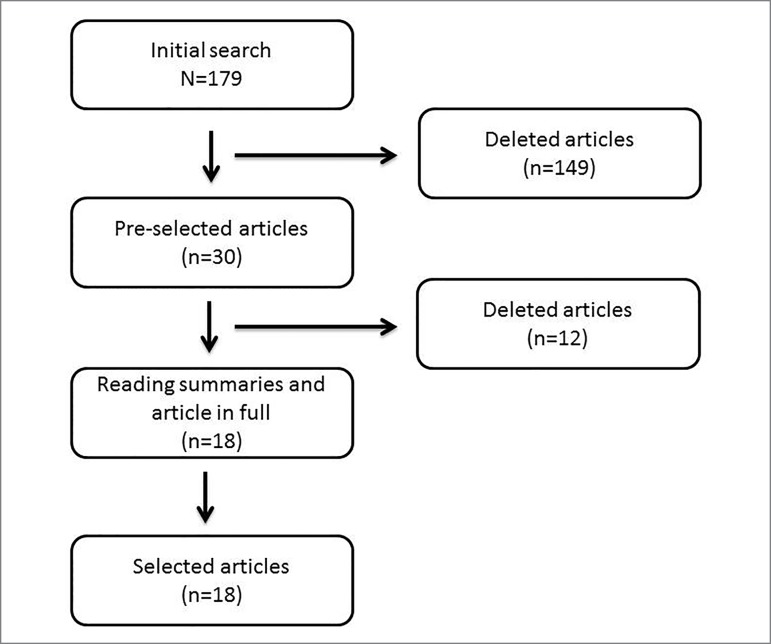
Flowchart of the steps followed to obtain the articles selected for this systematic review.

During the initial selection process 179 articles were found, of which 149 were excluded after reading the title, according to the selection criteria. The summaries were then read to check for compliance with the inclusion criteria and subsequently to confirm the eligibility of the article. Twelve studies were excluded. Finally, 18 papers were selected for analysis and discussion of results (Figure 1). Other articles were used to contextualize and discuss the studies presented.

## DISCUSSION

From the analysis of the studies included in this review, it is possible to verify that different genes have been investigated regarding the cardiovascular risk in HD individuals. Most of them are related to the inflammatory state and oxidative stress (OS) (Table 1) and vascular calcification (VC) (Table 2). However, genes related to left ventricular hypertrophy, dyslipidemia and hypertension have also been evaluated (Table 3).

**Table 1:** Polymorphisms in genes related to inflammation, oxidative stress and cardiovascular risk in hemodialysis subjects

Gene	Molecule	SNP	Methodology	Result	Reference
*SELE*	E Selectin, adhesion molecule	rs5355C > T	Cross-sectional study. 40 subjects (median age: 45 years; 50% women) and 30 controls (median age: 36.5 years; 63.3% women). SNP determination by PCR-RFLP	There was no difference in comparing the right and left media-intima thickness and the right and left cross-sectional areas between the CC, CT and TT from the do SELE SNP rs5355C > T	Isaac *et al*., 2014^40^
*HMOX1*	Heme oxigenase 1	The allele frequencies of the dinucleotide-guanosinatimidine repetitions length (the S allele represents shorter repetitions (< 27) and the L allele represents longer repetitions (≥ 27).	Cohort study. 1080 subjects (51.1% men; age: 59 years) and 365 controls (52.1% men; age: 57 years). SNP determination by PCR	The L/L genotype had higher mortality by CVD and by all causes.	Chen *et al*., 2013^45^
*ICAM-1*	Intercellular –I adhesion molecule	K469E; TT, TC and CC genotypes	Cross-sectional study. 1016 Caucasian subjects (656 with CVD: age 62.8 ± 15 59.9% men and 360 without CVD: age: 56.8 ± 14.3, 51.9% men); 824 controls (age: 52.1 ± 14; 55.2% men). SNP determination by PCR + allele-specific.	Upon stratifying the patients according to CVD clinical characteristics, there was a trend for higher frequencies of the T allele and TT genotype in patients with AMI; Bearing the T allele was an independent risk factor for CVD susceptibility.	Buraczynska *et al*., 2012^20^
*IL-6*	Interleukin-6	-634C/G, -174G/C and -572C/G.	Cross-sectional study 216 subjects with CAD (age: 58.6±10.6 years; 61.1% men). SNP determination by PCR RFLP.	Positive association of the -634GG and -174CC genotypes and risk of cardiovascular events; There was no association between the 572C/G SNP and the risk of cardiovascular events.	Song *et al*., 2015^8^
*IL-6, IL-10 and the tumor necrosis factor*	Interleucina-6 and 10 and the tumor necrosis factor	TNF:-308 G/A (rs 1800629); IL-6: -174 G/A (rs 1800795); IL-10: -1082 G/A (rs1800896)	Cross-sectional study. 169 Caucasian subjects (age: 62 ± 11 years; 62.1% men). SNP determination by PCR.	Heterozygotes for the *IL-10* gene had less cardiovascular events; Patients with the A allele (*TNF* gene) had higher risk of developing cardiovascular events Having the G allele (*IL-6* gene) had a protective effect over cardiovascular events.	Tosic Dragovic *et al*., 2016^17^
*NOX*	NADP oxidase	C242T	Cross-sectional study. 289 Chinese subjects: 192 without CVD (Group N; Age: 53.3 ± 12.6; 51.6% men) and 97 with CVD (Group D; age 54.4 ± 11.5 years; 51.4% men). SNP determination by PCR RFLP.	T+TT genotype frequency was significantly lower in the D Group when compared to the N Group.	Tang *et al*., 2010^29^
*RAGE*	Advanced glycation final products receptor	-374 T/A	Case control study 1866 Caucasian subjects (age: 61.6 ± 17.2; 57.0% men) and 1143 healthy subjects (age: 54 ± 19.1; 55.2% men); 63 subjects with ischemic stroke (age: 66.3 ± 14.5 years; 52.1% women). SNP determination by PCR.	Stroke subjects had lower frequencies of the A allele when compared to those patients without CVD.	Buraczynska *et al*., 2015^25^
*UGT1A1*	Uridine diphosphato-glucuronosiltransferase	UGT1A1*28	Cohort study. 661 subjects (50.7% men; age: 58 years) and 152 controls (53.9% men; age: 59 years). SNP determination by PCR.	Subjects with the 7/7 genotype had significantly higher bilirubin levels when compared to those with the 6/6 and 7/6 genotypes; Subjects with the 7/7 genotype had approximately 1/10 of the risk for cardiovascular events and 1/4 of the risk for all-cause mortality when compared to those bearing the allele 6.	Chen *et al*., 2011^37^

CAD: coronary artery disease; CVD: cardiovascular disease; HD: hemodialysis; HDL-c: high-density lipoprotein; *HMOX1*: heme oxigenase 1; *ICAM-1*: intercellular -1 adhesion molecule; IL-6: interleukin 6; IL-10: interleukin 10; NOX: NADP oxidase; PCR: polymerase chain reaction; PCR-RFLP: polymerase chain reaction - restriction-fragment length polymorphism; RAGE: receptor of advanced glycation end-products; SELE: E-selectin; SNP: single nucleotide polymorphism; UGT1A1: uridine diphosphate-glucuronosiltransferase.

**Table 2:** Gene polymorphism associated with vascular calcification and cardiovascular risk in hemodialysis individuals

Gene	Molecule	SNP	Study/Sample	Result	Reference
*ANRIL*	Non-codifying antisense RNA	rs10757278, rs4977574, rs10757274 and rs6475606	Cohort study. 284 subjects (age: 56.0 ± 2.0; 59.9% women). SNP determination by PCR.	Homozygote subjects for the risk allele (GG) from SNP rs10757278 had twice the risk of developing adverse cardiovascular event than those with the protective allele (AA or AG), even after adjusting for other risk factors such as *diabetes mellitus.*	Arbiol-Roca *et al*., 2017^81^
*MTHFR*	Methylenotetrahydropholato redutase	C677T	Cross-sectional study. 152 subjects (age: 56.8 ± 13.8; 54.6% men). SNP determination by PCR.	Higher VC score in subjects with the TT genotype than those with CC and CT; Higher prevalence of peripheral vascular disease with the polymorphism for all the individuals; Higher incidence of stroke with the polymorphism in young subjects (≤ 60 years); Positive association of CT and TT genotypes and VC.	Lee *et al*., 2011^73^
*Matrix Gla protein*	Matrix Gla protein	T-138C (rs1800802) G-7A (rs1800801)	Cohort study. 134 subjects. SNP determination by PCR.	VC progression velocity in subjects with the CC genotype. In the CC genotype subjects it was slow than in the CT or TT subjects; CT/TT genotype association with advanced age upon HD onset, male gender, high concentrations of calcium X phosphorus and LDL-c, low concentrations of HDL-c and ferritin and no use of angiotensin II receptor blockers with VC progression.	Yoshikawa *et al.*, 2013^68^
*VKORC1*	Epoxid-redutase Vitamin K	C1173T and G-1639A	Cross-sectional study. 54 subjects (age: 40.1 ± 12.5 years; 54% women). SNP determination by PCR.	Association between C1173T polymorphism and VC; The T allele was associated with higher likelihoods of VC and CVD clinically evident; The G-1639A polymorphism was not associated with VC and did show lower prevalence of clinically evident CVD.	Osman *et al.*, 2016^7^

PVA: peripheral vascular accident; VC: vascular calcification; HDL-c: high-density lipoprotein; VKOR: epoxid-redutase vitamin-K; LDL-c: low-density lipoprotein; MTHFR: methylenotetrahidrofolato reductase; PCR: polymerase chain reaction; SNP: single nucleotide polymorphism.

**Table 3:** Gene polymorphisms associated with dyslipidemia, arterial hypertension, left ventricular hypertrophy and cardiovascular risk in hemodialysis subjects.

Gene	Molecule	SNP	Study/Sample	Results	Reference
CTGF2	Connective tissue growth factor	G-945C	Cross-sectional study. 99 Caucasian subjects (age: 64 ± 13 years, 64% men). SNP determination by PCR.	Positive association between the GG genotype in cardiovascular events (CVA and MI) and CVD mortality.	Cozzolino *et al*., 2010^78^
ACE	Angiotensin converter enzyme	I/D	Cross-sectional study. 196 subjects (56,6% men; age: 62,3 ± 11.4 years). SNP determination by PCR RFLP.	Higher incidence of left ventricular hypertrophy and peripheral vascular disease in subjects with the D allele; Association between polymorphism and CVA incidence and hyperlipoproteinemia.	Tošić *et al*., 2014^58^
PPAR γ	Receptors activated by peroxisome proliferator	Pro12Ala and C161T	Cross-sectional study. 99 Chinese subjects (age: 60.2 ± 11.9 years; 53.5% men) and 149 controls (age: 51.7 ± 15.9 years; 56.4% men). SNP determination by PCR RFLP.	PC and CIMT in subjects with the CT + TT or Pro12Ala were lower than in the subjects with CC or Pro12Pro; CIMT of the Pro12Ala-CT161 subgroup was lower than in the Pro12Pro-CC161 and Pro12Pro-CT161 subgroups; CP of subgroup Pro12Ala-CT161 was lower than in the Pro12Pro-CC161 subgroup.	Liu *et al*., 2014^9^
VDR	Vitamin D receptor	BsmI	Cross-sectional study. 182 Caucasian subjects (57.1% men); 175 healthy subjects. SNP determination by PCR.	Direct association between the number of B alleles and LVMI, independently of treatment with anti-hypertensive and calcitriol; The number of B alleles was positively associated with LVMI changes.	Testa *et al*., 2010^75^
		BsmI	Cross-sectional study. 80 subjects (66.3% men; age: 57.3 ± 10.6 years); 40 healthy controls (65% men; age: 56.5 ± 11.2 years). SNP determination by PCR RFLP.	Subjects with the BB genotype had lower serum concentrations of 25-hidroxi vitamin D in comparison with the Bb and bb genotypes; The number of B alleles was positively correlated with the LVMI, but not with the intima-media thickness.	El-Shehaby *et al*., 2013^76^
SIRT 1	Sirtuin 1	rs7895833, rs7069102 e rs2273773	Cross-sectional study. 219 Japanese subjects (54.3% men; age: 60.4 ± 13.3 years); 803 control subjects (65.1% women; age: 61.3 ± 10.3 years). SNP determination by PCR.	TC and LDL-c serum concentrations were higher among bearers of the G allele (rs7069102) in comparison with the CC genotype in males; Coronary artery calcification scores were higher among bearers of the C allele (rs2273773) among all the individuals and in males.	Shimoyama *et al*., 2012^51^

ACE: angiotensin converting enzyme; CVA: stroke; TC: total cholesterol; CTGF2: connective tissue growth factor; CIMT: carotid intima-media thickness; HD: hemodialysis; MI: myocardial infarction; LVMI: left ventricular mass index; LDL-c: low density lipoprotein; CP: carotid plaque; PCR: polymerase chain reaction; PCR-RFLP: polymerase chain reaction - restriction fragment length polymorphism; PPAR: Peroxisome proliferator-activated receptors; SIRT 1: sirtuin 1; SNP: single nucleotide polymorphism; VDR: vitamin D receptor.

The selection of such genes is due to the fact that the presence of chronic inflammation and OS play an important pathogenic role in the development of CVD in HD individuals.^11^ The genes evaluated in relation to the inflammatory state and OS were tumor necrosis factor (TNF), interleukin-10 (IL-10), IL-6, intercellular adhesion molecule-1 (*ICAM-1*), transmembrane receptor for advanced glycation end products (RAGE), NADPH oxidase, uridine diphosphate glucuronosyltransferase (UGT1A1), E-selectin and heme oxygenase 1 (HO1) (Table 1).

TNF is one of the most relevant proinflammatory cytokines in the development, progression and complication of atherosclerosis, by reducing the expression of synthase endothelial nitric oxide, leading to endothelial dysfunction. TNF is positively regulated in progressive renal disease.^12^ Its gene is located on chromosome 6, being highly polymorphic in the promoter region. Most SNPs have the G/A substitution, and the most investigated is the -308 position. In the general population, the -308 G/A polymorphism is associated with elevated TNF production in AA homozygotes.^13^

IL-10 has been considered one of the most important anti-inflammatory and antiatherogenic cytokines. As it is mainly eliminated by the kidneys, its half-life is increased in HD individuals, leading to increased plasma concentrations.^15^ Furthermore, because of chronic monocyte activation, uremic patients produce larger amounts of IL-10 compared to healthy individuals. The IL-10 gene is located on chromosome 1, and the polymorphic sequences have been described in the promoter region at -592 C/A, -818 C/T and -1082 G/A positions. The -1082 G allele appears to be the most important, since G/G genotype producers 30% more cytokine, while low A/A genotype production is associated with increased cardiovascular mortality in HD individuals.^16^

IL-6 is a multifunctional cytokine involved in several contradictory processes as it has pro and anti-inflammatory effects and may promote atherosclerosis and muscle loss. Different haplotypes in the IL-6 gene can determine the levels of its transcription. The *IL-6* gene is located on chromosome 7p21 and has several polymorphisms in the promoter regions (-174 G/C, -634 C/G, -572 G/C and -597 G/A)^17^ associated with increased risk of CVD.

In this context, Song et al.^8^ published that genotypes IL-6-634GG and IL-6-174CC were associated with a higher risk of cardiovascular events in HD individuals. Nonetheless, Tosic Dragovic et al.,^17^ in addition to IL-6, also evaluated polymorphisms in IL-10 and TNF and concluded that cardiovascular morbidity could be under the influence of genetic polymorphisms in these cytokines.

Furthermore, *ICAM-1*, a cell surface glycoprotein, is a member of the immunoglobulin superfamily of adhesion molecules, responsible for the adhesion of circulating leukocytes to the activated endothelium, which is one of the first events in the pathogenesis of atherosclerosis. *ICAM-1* is expressed in the vascular endothelium, smooth muscle cells, macrophages and activated lymphocytes. Its expression can be positively regulated by inflammatory mediators.^18^

The *ICAM-1* gene is located on chromosome 19p13 and consists of seven exons. The polymorphism in which cytosine is replaced by thymine in the sixth exon of that gene results in the substitution of the glutamic acid (E) for lysine (K) in the immunoglobulin domain 5 of the *ICAM-1* protein (K469E). This polymorphism is involved in inflammatory diseases and atherosclerosis.^19^ In HD individuals, being a carrier of the T allele of this polymorphism was considered a risk factor regardless of susceptibility to CVD.^20^

RAGE is a member of the immunoglobulin superfamily, which recognizes a wide range of endogenous ligands that accumulate in tissues during aging, chronic degeneration and inflammation. RAGE expression is low under normal conditions, whereas in pathogenic conditions, such as diabetes or inflammation, it is associated with increased expression.^21^

The gene encoding RAGE is located on chromosome 6p21^3^ in the major histocompatibility complex (MHC), and comprises 11 exons. Of all the polymorphisms identified in this gene, the -374 T/A variant was associated with CVD.^22^ Several studies have shown a strong link between the genotype -374A or AA and protection against vascular disease.^23^^,^ 24 These results were also confirmed in Caucasian individuals in HD, in which the presence of the A allele of this polymorphism had a protective effect against cerebrovascular accidents.^25^

NADH/NADPH oxidase is a membrane-associated enzyme that produces superoxide in vascular and endothelial smooth muscle cells,^26^ being the most important source of reactive oxygen species in intact arteries.^27^ The CYBA C242T polymorphism in this enzyme is associated with increased production of superoxide in blood vessels in individuals with CVD.^28^ Indeed, in HD subjects, CT + TT genotypes were considered independent protection factors for CVD, indicating that the presence of this polymorphism is a significant factor in CVD development.^29^

Bilirubin has antioxidant and anti-inflammatory properties and its antioxidant and antiatherogenic effects are believed to be due to its ability to inhibit the oxidation of LDL (low density lipoprotein) and other lipids,^30^ to eliminate free radicals^31^ and neutralize OS.^32^ Studies have shown an inverse association between serum concentrations of bilirubin and coronary and peripheral vascular disease and stroke.^33^^,^ 34

Serum bilirubin concentrations are controlled by the UGT1A1enzyme, which contributes to the bilirubin glucurinidation and, consequently, is the main determinant of its clearance in humans. A common cause of decrease in UGT1A1 activity is the insertion of a TA into the TATAA box in the promoter region of the *UGT1A1* gene, designated UGT1A1*28.^35^

Individuals homozygous for 7 repetitions (7/7) have higher serum bilirubin concentrations than heterozygotes (7/6) or those with the wild type 6-repetitions (6/6).^35^^,^ 36 In HD patients, UGT1A1*28 polymorphism showed a strong effect on bilirubin levels and genotype 7/7 appears to have a significant effect on the reduction of cardiovascular events and death.^37^

However, these studies related to polymorphisms in these genes were performed with small sample sizes. Thus, it is suggested the replication of these associations found in other cohorts of patients, possibly by different strategies, validate the results and clarify the role of these genes in CVD in the context of a uremic environment.

E-selectin, an 11 kDa cell surface glycoprotein, is an adhesion molecule of the selectin family, which recruits circulating leukocytes through adhesive interactions and participates in cell signaling and bearing, which in turn leads to a firm adhesion.^38^ E-selectin is not detected in inactive endothelial cells but it is synthesized rapidly in response to certain cytokines and other pro-inflammatory stimuli, making it a marker of the "activated" endothelial phenotype.^39^

Issac et al.^40^ evaluated the difference in plasma pregnancy-associated protein A (PAPP-A) levels between the rs5355C> T genotypes in the E-selectin gene and also investigated the possible association between serum PAPP-A and this polymorphism with blood pressure and lipid profile in HD subjects. There was no direct association between polymorphism, serum PAPP-A concentration and intima-media thickness. The authors suggest that this association with carotid atherosclerosis may reflect an indirect mechanism of both polymorphism and serum PAPP-A levels with cardiovascular risk factors, blood pressure and HDL-c (high density lipoprotein), rather than a direct effect on the vasculature.^40^

PAPP-A is produced mainly by the syncytiotrophoblast during gestation, but also by fibroblasts, osteoblasts, and vascular endothelial and smooth muscle cells, in both men and women. It is suggested that elevated serum PAPP-A concentrations may be a marker of the degree of echogenicity of atherosclerotic lesions in the carotid arteries.^41^

*HMOX1* is a cytoprotective enzyme that potentially exerts antioxidant, anti-inflammatory, antiapoptotic and angiogenic functions through its reactive products.^42^ The *HMOX1* gene was mapped on chromosome 22q12^43^ and the number of guanosine thymidine dinucleotide [GT)_n_] repeats in the promoter region of this gene is inversely associated with HO1 mRNA (messenger ribonucleic acid) levels and the activity of the transcribed enzyme.^44^ In fact, HD individuals with longer lengths (GT)_n_ in this gene had greater inflammation and OS, and are at greater risk for cardiovascular events in the long term, as well as being more susceptible to mortality.^45^

Dyslipidemia is an important risk factor for the development of atherosclerotic lesions.^46^ Thus, the genes evaluated for the presence of polymorphisms that could influence the onset of CVD were sirtuin 1 (SIRT1) and PPARγ (Table 2).

SIRT1 acts on endocrine signaling, specifically on glucose and fat metabolism,^47^^,^ 48 through the activation of α and β proteins at the liver X receptor, which regulate lipid metabolism.^49^ In adipose tissue, SIRT1 interacts with the PPARγ, inhibiting transcriptional activity, and consequently adipogenesis.^47^ Thus, SIRT1 is associated with lipid metabolism, and polymorphisms in its gene may affect the lipid profile. This association was observed in Japanese individuals in HD, in whom the presence of the rs7069102 and rs2273773 polymorphisms was associated with abnormal cholesterol metabolism and coronary artery calcification, respectively, especially in men.^50^

In turn, PPARγ is a nuclear hormone receptor that regulates the target genes responsible for lipid and glucose metabolism, inflammation, proliferation and necrosis of tumor cells, organ sclerosis and fibrosis.^51^ Because it acts on the regulation of metabolism and inflammation, it can affect atherosclerotic processes.^52^

Numerous genetic variations of the gene encoding PPARγ influence its regulatory role in gene transcription.^9^ The most common SNPs are Pro12Ala and C161T. The Pro12Ala polymorphism is characterized by a CG substitution on the B exon, resulting in the conversion of proline to alanine at residue 12 of the protein. The other is CT replacement at the position of nucleotide 161 at exon 6 (C161T).^53^ Previous studies have shown that these polymorphisms may play an important role in carotid artery atherosclerosis in populations characterized by dyslipidemia, diabetes, obesity and CVD.^54^^,^ 55 However, in Chinese HD individuals, these two polymorphisms were associated with significant risk factors for CVD, such as increased C-reactive protein and carotid intima-media thickness, as well as formation of atheromatous plaques in these arteries, but not to the lipid metabolism and nutrition.^9^

The presence of systemic arterial hypertension leads to an increased risk of fatal and non-fatal cardiovascular events.^56^ In this sense, a study included in this review investigated the influence of polymorphisms in the angiotensin converting enzyme (ACE) gene on cardiovascular morbidity in HD individuals^58^ (Table 2).

ACE converts inactive angiotensin I into its active form, angiotensin II, a potent vasoconstrictor and the main product of the renin-angiotensin system.^58^ The ACE-encoding gene is located on the long arm of chromosome 17 and comprises 26 exons and 25 introns. A polymorphism found in this gene is the insertion (I)/deletion (D), the deletion is considered a mutation. There are three different I/I, I/D and D/D genotypes, and each can influence ACE activity. The highest levels of plasma ACE are found in DD homozygotes. Homozygotes with genotype I/I have the lowest levels, and I/D heterozygotes have intermediate plasma levels of this enzyme.^59^ This polymorphism leads to a greater predisposition to the development of CVD, such as myocardial infarction, stroke and other atherosclerotic disorders.^60^^,^ 61 Indeed, in HD individuals, the ACE gene polymorphism was associated with the development of stroke, and the D allele of this gene significantly increased the risk of developing left ventricular hypertrophy and peripheral vascular disease. However, the authors suggest the need for a longer follow-up to reach a definitive conclusion about the influence of this polymorphism on cardiovascular morbidity and its importance in daily clinical practice.^57^

VC is highly prevalent among CKD patients, progressing often over a relatively short period of time, and is a strong predictor of CVD and all-cause mortality in this population.^62^^,^ 63 Thus, studies included in this review, three investigated polymorphisms in genes that could influence VC and, consequently, cardiovascular risk, being: matrix Gla protein (MGP), vitamin K epoxide reductase (VKORC) and 5,10 methylenetetrahydrofolate (MTHFR) (Table 3).

MGP is a vitamin K-dependent protein with 84 amino acids and a molecular weight of 12 kDa.^64^ It is suggested that this would be a critical factor in the development of atherosclerosis in HD individuals.^65^ The gene encoding MGP has several SNPs in the its promoter and coding regions.^66^ In particular, the MGP-138CC genotype of the T-138C polymorphism in the gene of this protein may be associated with a slower progression of VC in HD patients. Thus, the authors propose that the genotype of the MGP gene may be a genomic biomarker predictive of VC progression. In addition, this unalterable biomarker may be useful in disease detection and classification, treatment response prediction, treatment efficacy, and prognosis.^67^

Still within this context of VC, Osman, El-Abd and Nasrallah^7^ investigated the association of polymorphisms in the VKORC1 gene with CVD in HD individuals, by the presence of clinically evident CVD and/or VC. The authors found that polymorphisms in this gene were associated with prevalent cardiovascular calcification and clinically evident CVD, with patients with the C1173T polymorphism being at higher risk of disease and those with G-1639A, a lower risk. However, these results need to be confirmed in studies involving the measurement of carboxylated vitamin K, MGP and coagulation factors for better interpretation.^7^

In fact, VKOR is responsible for the recycling of vitamin K, which need in the human body is very low. The inactivation of this enzyme increases the vitamin's requirements to values above the one present in the diet, resulting in its functional insufficiency.^68^ VKORC1 is the gene coding for VKOR, and polymorphisms in this gene were associated with the availability of vitamin K active for the carboxylation of coagulation factors, particularly resistance to coumarin.^69^ Increased concentrations of coagulation factors associated with these polymorphisms may be related to vascular events as a consequence of hypercoagulability.^70^

5,10 MTHFR is one of the major enzymes involved in the metabolism of homocysteine, which has atherogenic properties in the blood vessels. Mutations in the MTHFR gene could reduce its enzymatic activity and cause hyperhomocysteinemia, which is a risk factor for atherosclerosis due to endothelial dysfunction and OS.^71^ In HD individuals, there was a strong relationship between the presence of the C677T polymorphism in the MTHFR gene and VC, as compared to the CC genotype, patients with CT and TT genotypes had VC adjusted odds ratios of 1.39 and 1.58, respectively (*p* < 0.005).^72^

Left ventricular hypertrophy is one of the most important risk factors for all-cause and cardiovascular mortality in HD individuals.^73^ Two studies included in this review investigated the effect of polymorphisms in the vitamin D receptor gene on left ventricular hypertrophy and, consequently, on the cardiovascular risk this population^74^^,^ 75 (Table 2).

Vitamin D deficiency is common in these patients and may have significant health consequences.^76^ Myocardium is an important vitamin D target, and three common polymorphisms (BsmI, ApaI and TaqI) at the 3' end of the vitamin D receptor have been intensively investigated. In this sense, Testaet al.^74^ and El-Shehaby et al.^75^ observed that, in patients on dialysis, the B allele of the BsmI polymorphism in the vitamin D receptor gene was independently related to left ventricular hypertrophy and is associated with a greater rate of its progression. In addition, the B allele of this polymorphism may be considered a novel marker of alteration of vitamin D signaling in these patients.

In addition, a study included in the present review investigated the influence of polymorphism in the connective tissue growth factor (*CCN2*) gene on cardiovascular morbidity and mortality in HD subjects^77^ (Table 2). *CCN2*, a prophylactic cytokine secreted by human endothelial cells, is involved in atherogenesis, since its mRNA is expressed in smooth muscle cells of atherosclerotic blood vessels, but not in normal homologous arteries.^78^

In addition, *CCN2* protein expression is significantly greater in atherosclerotic plaques compared to fibrous plaques, more stable, and may increase monocyte migration in atherosclerotic lesions, thus contributing to atherogenesis.^79^ In Caucasian individuals in HD, polymorphism in *CCN2* gene was considered a prognostic risk factor for cardiovascular morbidity and mortality.^77^

The authors of this study suggest that these results may have important implications for a better understanding of the link between accelerated atherosclerosis and increased mortality in this population.^77^

Finally, individuals homozygous for the risk allele (GG) of the SNP rs10757278 in the ANRIL (antisense non-coding RNA) showed a two-fold increased risk of adverse cardiovascular event than those with the protective allele (AA or AG), even after adjustment for other risk factors such as diabetes *mellitus*.^80^ ANRIL is located on chromosome 9p21.3 and is considered the genetic factor most strongly associated with atherosclerotic CVD.^81^ Increased expression of this gene speeds up proliferation, increases adhesion, and decreases apoptosis,^81^ mechanism related to the pathogenesis of atherosclerosis.

## CONCLUSION

Overall, the results of the studies included in this review suggest that polymorphisms in genes related to inflammation and OS and VC affect cardiovascular risk in HD individuals. In addition, polymorphisms in genes considered risk factors for CVD, such as dyslipidemia, arterial hypertension and left ventricular hypertrophy, also influence cardiovascular morbidity and mortality in this population.

## LIMITATIONS AND PERSPECTIVES

The studies included in this review had as limitations the use of small sample size and specific ethnic groups, and it is not possible to extrapolate the results to HD patients in general. Most of the studies are of the transversal type, making it impossible to verify the cause-effect relationship of the presence of a specific allele. In addition, the studies generally analyze polymorphisms in a single gene, and haplotype analysis in some cases is more interesting, that is, the analysis of polymorphisms in genes close to the analyzed one that could influence the risk of developing the disease. Finally, it should be considered that the only influence of the polymorphism on the risk of developing the disease is small, since environmental factors, lifestyle and, in the case of HD individuals, the presence of other comorbidities (such as kidney disease, diabetes *mellitus*, hypertension, among others) may interact with the polymorphism, influencing the phenotype determination.

Despite the aforementioned limitations, it is known that evaluating the presence of these polymorphisms is extremely important, since the identification of patients with high-risk genotypes may enable early preventive strategies and provide a closer follow-up of the appropriate target populations. In addition, it is essential to recognize a predictive biomarker for morbidity and mortality and to have better identification of high-risk groups. Finally, nutrigenetics, a science that studies the effect of genetic variation among individuals in response to diet, is an important aspect to consider, since, in the context of a personalized recommendation, the knowledge of this gene-nutrient interaction indicates which individuals could benefit from adopting a specific diet. It also points out that other environmental factors may interfere with the gene-nutrient relationship.
